# Causal thinking and causal language in epidemiology: it's in the details

**DOI:** 10.1186/1742-5573-2-8

**Published:** 2005-07-29

**Authors:** Robert Lipton, Terje Ødegaard

**Affiliations:** 1 Research Scientist, Prevention Research Center, 1995 University Ave. Suite 450, Berkeley, CA 94704, USA; 2 Faculty of Health and Social Work, Lillehammer University College, N-2626, Lillehammer, Norway

## Abstract

Although epidemiology is necessarily involved with elucidating causal processes, we argue that there is little practical need, having described an epidemiological result, to then explicitly label it as causal (or not). Doing so is a convention which obscures the valuable core work of epidemiology as an important constituent of public health practice. We discuss another approach which emphasizes the public health "use value" of research findings in regard to prediction and intervention independent from explicit metaphysical causal claims. Examples are drawn from smoking and lung cancer, with particular focus on the original 1964 Surgeon General's report on smoking and the new version released in 2004. The intent is to help the epidemiologist focus on the pertinent implications of research, which, from a public health point of view, in large part entails the ability to predict and to intervene. Further discussion will center on the importance of differentiating between technical/practical uses of causal language, as might be used in structural equations or marginal structural modeling, and more foundational notions of cause. We show that statistical/epidemiological results, such as "smoking two packs a day increases risk of lung cancer by 10 times" are in themselves a kind of causal argument that are not in need of additional support from relatively ambiguous language such as "smoking causes lung cancer." We will show that the confusion stemming from the use of this latter statement is more than mere semantics. Our goal is to allow researchers to feel more confident in the power of their research to tell a convincing story without resorting to metaphysical/unsupportable notions of cause.

## Introduction

### Causal thinking and causal language in epidemiology

A primary goal of epidemiological research is the ability to determine how exposures are related to outcomes. We are interested, at the population level, in what caused the cancer, the heart attack, the cholera epidemic or the food poisoning. Our methods have developed rapidly over the last four decades to account for, among other things, confounders, retrospective and longitudinal data, and bias. In an effort to systematize the causal enterprise, similar to efforts in other relatively young fields of scientific inquiry, epidemiologists have sought to tie such methods to an overarching causal rubric such as Popperian falsification, Mill's analysis of causation in terms of necessity and sufficiency, *ceteris paribus *conditions/control of confounding [1] and/or counterfactuals. Such efforts, while being very useful in advancing the field and providing guidance for understanding exposure and outcome relationships, have tended to ignore the claim of Hume, among other writers, that causal connections cannot be observed or objectively proven. Thus, on the one hand, a great deal of effort is spent to carefully develop methods aimed at revealing causal relationships, while on the other we are being told – rather persuasively – that we cannot ultimately determine causal relationships, or that we should refrain from attempts to establish causal relationships because these should be understood as different from nomological or probabilistic relations. Obviously, these tensions have not stopped scientific, let alone epidemiological, efforts from proceeding apace (nor should they).

Nevertheless, fundamental issues bearing on how the relationship between exposures and outcomes are assessed, interpreted and discussed, are left more ambiguous than necessary. And to be clear, this is not just a theoretical issue, since such ambiguity allows for real world problems to arise that, with a small amount of care, are easily avoided. In this essay, we will explore some limitations on obtaining causal information, and on how such epidemiological information should be disseminated, both to lay and professional audiences, in a more useful and less confusing manner than is often presented. The goal of our argument is to invite a less anxious and more humble, yet forceful, approach toward assessing epidemiological research. This approach will show that the process of examining exposures and outcomes is the important factor, in service to prediction and intervention, not an illusory ability to identify and articulate apparently more fundamental causal connections.

## Analysis

### Public health issues and causation-speak in the 2004 Surgeon General's report

Among the possible reasons so much has been written about causation and epidemiology is that in significant ways epidemiology is a science, and as such is definitionally interested in causation. If there is a shared discourse in epidemiology as a field it revolves around the manner in which exposures are related to outcomes in service to analyzing truly pressing public health issues.

Recently, a new Surgeon General's report on smoking, responding to and expanding on the original 1964 report, included a section explicitly discussing issues of causal claims and providing guidelines for determining the strength of causal relations [2]. The new report quotes the original 1964 report: "after vigorous discussions they could neither precisely define nor replace the word 'cause,' a reflection of the same problem that philosophers have confronted over the centuries." Further, the 1964 report noted that

when a relationship or an association between smoking ... and some condition in the host was noted, the significance of the association was assessed. The characterization of the assessment called for a specific term .... The word cause is the one in general usage in connection with matters considered in this study, and it is capable of conveying the notion of a significant, effectual relationship between an agent and an associated disorder or disease in the host. Granted that these complexities were recognized it is to be noted clearly that the Committee's considered decision to use the words ' "a cause" or "a major cause" or "a significant cause" or "a causal association" ' in certain conclusions about smoking and health affirms their conviction. [3] (p. 21)

The authors of the new report correctly point out that while the original report is quite useful and serves as one of the most important examples of comprehensive assessment of exposures and outcomes in public health history, there is some level of confusion associated with the language of causation. Indeed, the 1964 report is clearly struggling to articulate and justify its use of causal terms; e.g., in the passage quoted, the strained language of the last sentence is revealing. Circularity threatens when a choice of terminology for the purposes of describing one's findings is justified in part by a wish to "affirm convictions" in regard to the findings. Are we to suppose that the findings do not speak sufficiently eloquently for themselves?

In an attempt to address and even regiment the use of causation-speak, the authors of the new report, in addition to providing a very comprehensive list of causal statements related to smoking and health outcomes, discuss what they consider to be a less confusing approach towards using causal language and ascribing cause in epidemiology. Unfortunately, their efforts, while clearly useful as a guide to assessing possible implications of research, beg the question of whether explicit causal language is really needed in presenting and discussing research in the first place. Other begged questions concern how such causal language is *necessarily *linked to the substantive research and how hierarchies of causal strength are to be determined.

Starting on page 11 of the new report [2], their careful listing of causal statements from previous reports is strikingly idle in view of the fact that in many of the statements in the new report there is no explicit use of the word "cause" when these succinctly describe the current state of research. Examples are: "Autopsy studies suggest that cigarette smoking is associated with a significant increase in arteriosclerosis of the aorta and coronary arteries," "Recent autopsy studies confirm that pulmonary emphysema is much more frequent and severe in cigarette smokers than nonsmokers," or "Women cigarette smokers experience an increased risk for subarachnoid hemorrhage."

These statements can be contrasted with others such as "It is also more prudent to assume that the established association between cigarette smoking and coronary disease *has causative meaning *than to suspend judgment until no uncertainty remains" (p. 13, our emphasis). The discomfort on display in the last sentence is clear: responsible prudence apparently dictates the use of explicit causal language even though the findings, on their own, i.e. "established association" can be taken to be a meaningful statement of cause (more on this below). There is, further, an implied and somewhat ambiguous assumption that complete certainty, although not available here, is theoretically achievable, yet not needed, for causal information to be conveyed. Although this latter point is one with which we can strongly agree, we nevertheless argue that worrying about complete certainty is not useful for the simple reason that this level of certainty is not available. Indeed, if the information obtained from the "established associations" allows for effective prediction and/or intervention, then it is not clear what other information or language would be needed in terms of causal argumentation. Thus, the above remark seems to be intended to justify and/or motivate health policy (not necessarily a bad thing but off-point for the purposes of this discussion) rather than to improve our causal understanding of the relationship between smoking and coronary disease.

One problem is that, as Hume described and the authors confirm [2,4], while the use of causal language can be psychologically compelling, the causal nexus will never lend itself to be empirically detected or generally proven. Particularly in regard to the last statement from the Surgeon General's report, uncertainty will always remain. This uncertainty can be thought of as supporting a more probabilistic approach towards causation [5]. Parascandola and Weed point out that probabilistic models of causation are essentially more flexible than deterministic approaches. Their argument centers on the idea that since it is impossible to ever know all the constituent elements in a deterministic causal model, why not allow for some level of probabilistic ambiguity? The need to say anything definitive about this dichotomy, however, is not in the realm of the strictly scientific, nor is their discussion of what constitutes science and what constitutes public health policy, and why different notions of science might apply to the two. Although these are different contexts, the inability to "prove" or objectively "see" causation, however, still applies to both.

More importantly, a fundamental inability to determine cause is not necessarily a serious concern for epidemiologists because causal information can still be conveyed without getting bogged down in such epistemological and metaphysical issues. Thus, the struggle to develop a causal context relating tobacco to illness in the new report displays a level of anxiety that would be unnecessary if a more pragmatic approach toward causal information were used.

#### A short caveat on realism in science

We are not in this essay attempting to revisit the long-standing debate between realism and pragmatism in science. (A relatively current, although ultimately unconvincing, exploration of realism in epidemiology was discussed by Renton [6].) For the sake of making our argument, we accept the natural ontological attitude (NOA) developed by Arthur Fine [7] as being closely in line with our approach toward thinking about causation. His argument is, in fact, a generalization of what has been discussed here. That is, he asks what is the efficacy of having something be considered "real" in the same manner as something being determined as "causal." He is interested in the ability to manipulate the world, to predict and intervene. Being able to determine something as real, in a metaphysically emphatic sense, something he and we doubt can ever be accomplished, is beside the point when dealing with the actual process of doing science.

### An alternative approach toward causal thinking

Once a famous epidemiologist, K, stated that causation is easy, "smoking causes lung cancer," adding a sarcastic "it's obvious" shrug of his shoulders to emphasize his point. This was in answer to a naïve query regarding how certain we could be about ever saying that X causes Y. K's response was a catalyst to our interest in epidemiologists' use of causal language, both in the day-to-day workings of any particular epidemiological project and in the more extended long-range meta-discussions bearing on causal thinking in epidemiology. It is our suggestion that K's remark, while presumably intended to lend scientific weight to the findings that he had in mind, might rather have done them a disservice.

What, if anything, would underwrite an explicit causal claim, in this kind of context? We shall consider an admittedly not uncontroversial discussion of causality by G.E.M. Anscombe [8]. She identifies a claim shared by received philosophical views about causal connections as being either a kind of *necessary connection *between events, or as *instancing an exceptionless generalization *– a universal claim – saying that a certain kind of event will always be preceded by certain others: "If an effect occurs in one case and a similar effect does not occur in an apparently similar case, there must be a relevant further difference." [8] (p. 88)

Versions of the associations of causality with either necessity or universality are found throughout the history of philosophical thinking about the subject – Anscombe mentions Aristotle, Spinoza, Hobbes, Hume, Kant and Russell – although the accounts vary greatly with respect to whether the focus is on necessity or universality. Aristotle, Spinoza and Hobbes go for necessity, with the latter expressing the connection as being logically rather than naturally compelling, and so does Kant, who secured necessity (and also invariability) by introducing the idea of causality as a rule governing our very ability to understand sequences of events. Hume famously saw no necessity at work in the collision between two billiard balls. He described how the potential for experiencing the same thing repeatedly – an experience of "constant conjunction" – provided the basis for an irresistible, species-wide, but ultimately psychological idea of necessity. This was considered an essential but not empirically justifiable part of the complex idea of causality. Hume explained the latter as an upshot of experienced *exceptionless *generality. (Kant's view was of course motivated by the "scandalousness" of this "too disastrous to be true" position.) Russell, at one stage, argues that it is for universality to *explain *the notion of causal connections as being necessary. What they all share, however, is that causation is about necessity or universality or both.

Anscombe challenges this fundamental view; i.e., she challenges the shared claim quoted above:

... it's not difficult to show it *prima facie *wrong to associate the notion of cause with necessity or universality in this way. For, it being much easier to trace effects back to causes with certainty than to predict effects from causes, we often know a cause without knowing whether there is an exceptionless generalization of the kind envisaged, or whether there is a necessity. [8] (pp. 136–137)

Thus if one, for example, has been intimate with someone developing mononucleosis, one might expect to contract it, and if one does one would assume that one knows the cause, but no doctor would venture to bet on one's coming down with the disease if invited to do so before a diagnosis.

What is Anscombe's point? She proposes that we may have causal knowledge without having clarified what is involved in causation, in any of the heavy-duty philosophical senses discussed above:

Compare the possibility of wanting clarification of 'valency' or 'long-run frequency,' which yet have been handled by chemists and statisticians without such clarification; and valencies and long-run frequencies, whatever the right way of explaining them, have been known. Thus one of the familiar philosophic analyses of causality, or a new one in the same line, may be correct, *though knowledge of it is not necessary for knowledge of causes*. [8] (p. 136, our emphasis)

Moreover, both necessity and universality fail to focus, she argues, on something "so obvious as to seem trite," and proposes to replace the shared feature of accounts of causality given above with the following:

... causality consists in the derivativeness of an effect from its causes. This is...the common feature of causality in its various kinds. Effects derive from, arise out of, come of, their causes. For example, everyone will grant that physical parenthood is a causal relation. Here the derivation is material, by fission. Now analysis in terms of necessity or universality does not tell us of this derivedness of the effect; rather, it forgets about that. For the necessity will be that of the laws of nature; through it *we *shall be able to derive knowledge of the effect from knowledge of the cause, or vice versa, but that does not show us the cause as source of the effect. Causation, then, is not to be identified with necessitation. [8]

Causal claims thus assert something *other than *the claim that the effect would not have occurred if the cause had not occurred; rather, they say something about *how *the effect was brought about by the cause. It is her claim that the philosophical tradition, by not attending to this, misses out on something fundamental to causality.

But doesn't this also accurately characterize a kind of question that epidemiology is normally *not *in a position to answer, and that it is also *not *part of its typical purview? The epidemiologist might find himself at home in the philosophical tradition that Anscombe is concerned to put to one side, or that part of it which looks to strict or statistical laws when attempting to articulate the essence of causal relations, and choose to dismiss her attempt to refocus philosophical awareness of causation as irrelevant. But to the extent that he finds Anscombe's argument intriguing, even if not compelling, to that same extent he is faced with reasons to refrain from couching his findings in causal language. This is of course not to say that there couldn't be causal statements about the relationship between, say, smoking and cancer, presumably uncovered by scientists in the areas of physiology and medicine, but *they *would presumably venture to articulate precisely how the effect is brought about by the cause, in given cases.

Anscombe remarks that our knowledge of causality is acquired through the learning of diverse causal concepts associated with actions and events. If talk about causes shows the possession of a concept "cause," this is a sophisticated addition to the language of someone mastering other causal concepts:

... the word 'cause' can be added to language in which are already represented many causal concepts. A small selection: *scrape, push, wet, carry, eat, burn, knock over, keep off, squash, make *(e.g. noises, paper boats), *hurt*. But if we care to imagine languages in which no causal concepts are represented, then no description of the use of a word in such languages will be able to represent it as meaning *cause*. [8] (p. 137)

In an epidemiological vein, when we say for lung cancer that smoking increases risk by ten times compared to those who do not smoke, the causal language is "increases risk by ...." What is the purpose then of adding "and this is likely to be a causal relationship"? Some may point out that that one statement can stand in for the other, and we would agree (under very specific circumstances), but this begs the question as to why epidemiologists seem to need to privilege one type of causal utterance over another, or to redundantly use language that explicitly uses the word "cause." Further, there is actually an asymmetry here between types of causal utterances. Claiming that something "increases risk" is, for the most part, less ambiguous than saying something causes another. Thus, in practice, different kinds of causal statements are not necessarily substitutable.

### The knowledge of having hands

Readers with a surplus of philosophical patience might perhaps join us in also considering K's response against the background of G.E. Moore's famous attempt to argue that he had at least *some *bits of knowledge that were certain, and Wittgenstein's comments. Moore argued for the claim "I know I have two hands!" by first holding up one, while remarking, "Here is one hand," then the other [9,10]. This approach to knowledge, while intending to appear naïve, is actually under-girded by a sophisticated epistemological superstructure [11]. The thought was, roughly, that knowledge claims might be supported and skepticism about empirical knowledge refuted by providing examples of bits of such knowledge unquestionably available to a subject's mind in its perceptual encounter with his environment.

Wittgenstein was not convinced. His argument, much watered-down, went something like the following: let's say someone is playing a piano sonata, it is clearly a piece that is demanding two hands, but while he is playing he suddenly yells to his audience "I know I have two hands!" This phrase, apropos of nothing, is essentially meaningless; the knowledge of the two hands is, as it were, implicit in the playing of the sonata, the utterance of his claim to "know" this is idle. Indeed, if someone came up to you and said, completely out of the air, "I know I have two hands," there would be no context and no real information conveyed. He claimed in *On Certainty *[12] that Moore's attempt to justify his knowledge claim was misguided, and is interested in reinterpreting what is going on in such an example. He argues that although Moore's knowledge claims are indeed of a kind that it does not make sense to doubt, this is not because they are supported by irrefutable evidence:

The propositions, however, which Moore retails as examples of such known truths are indeed interesting. Not because anyone knows their truth, or believes he knows them, but because they all have a similar role in the system of empirical judgments. [12] (remark 137)

What is this "role"? Wittgenstein characterizes these claims metaphorically as, for example, what one can "discover ... like the axis around which a body rotates. ... [where] the movement around [the axis] determines its immobility" (remark 152), as the "rock bottom of one's convictions, ...one might almost say that these foundation walls are carried by the whole house" (remark 248), or "... the *questions *that we raise and our *doubts *depend on the fact that some propositions are exempt from doubt, are as it were like hinges on which those turn" (remark 341). These claims are then a kind of spin-off from the use of language, mistaken for empirical propositions, and treated by Moore as exempt from doubt. But,

I should like to say: Moore does not know what he asserts he knows, but it stands fast for him, as also for me; regarding it as absolutely solid is part of our method of doubt and enquiry. [12] (remark 151)

Wittgenstein was making the argument that knowledge is really not explicable unless tied to a process of doing things in the world. We know the pianist has two hands by virtue of his playing the sonata; the epidemiologist's research on exposures and effects, and his findings, make it impossible not to think of smoking as a cause of cancer, although the claim transpires rather than follows from the material.

### The redundancy of emphatic causation claims

Let's look at an example of the effect of introducing an emphatic causality-claim into an epidemiological context. Consider the differences between saying

(1) Smoking causes lung cancer,

(2) If you smoked 2 packs a day for X amount of years, your chance of getting lung cancer would be 10 times greater than a non-smoker,

and finally,

(3) If you smoked 2 packs a day for X amount of years, your chance of getting lung cancer was 10 times greater than a non-smoker and *it's causal! *(Perhaps stamping one's feet for emphasis.)

Statement (1) needs the information in statement (2) to be useful from an epidemiological or public health standpoint. Statement (2) describes (of course in a somewhat sketchy way) an increased risk (in itself a causal statement) associated with exposure. (Information about attributable risk could also be included.) Does any other information need to be conveyed, beyond such description, such as in statement (3)? What is the nature of this last statement? Is the addition of "*and it's causal*" to statement (3) based on the content of statement (2) in a manner that makes (3) into an articulation of something that necessarily follows from (2), although it is not articulated there? Or does "*and it's causal*" convey some additional information that is useful/necessary, and if so, on which grounds?

Although epidemiologists may think that being able to say very specifically that smoking causes lung cancer is an important part of the research process, this kind of claim would not be underwritten by research findings. One might speculate that being able to make an explicitly causal claim is a desideratum for the professional culture, a desire or inculcated need to be able to make use of explicit causal language when stating conclusions or findings. After all, the use of causal language for purposes of summarizing or concluding may allow others to quickly ascertain whether this research is worth paying attention to or not, depending on whether causal claims are being made. That such claims might also be policy-driven, rather than demanded by the research effort itself, need not be germane to such a cultural trait.

The causal work actually done (i.e., the useful scientific information) is rather embedded in the longer detailed description or story, and this does not necessarily have to include any explicit language involving claims about causation. What we can say, with absolute certainty (taking into account different variables), is that a specific association was found. We cannot, with similar certainty, say that a causal relationship was found, nor do we need to do so. The former claim is accurate to the extent that the research methods were good and repeatable, the latter, explicitly causal claim, may not even be capable of being assessed.

### More than semantics

This may seem, as mentioned before, to be just a semantic quibble, but it is not just that. Whether – as Hume argued – causation can neither be proven nor fully experientially justified, or the findings provide inadequate clues as to *how *the assumed effect was brought about by the assumed cause, we are in effect left with provisional approaches toward treating such relationships between exposures and outcomes [4] (pp. 4–10). To be clear, we are not arguing that people do not think in causal ways, but that when we try as epidemiologists very explicitly to say "X causes Y," we put ourselves in a position similar to that of G.E. Moore saying "I know I have two hands." The knowledge is in the doing; the causal information is in the explicit explanation of how smoking is related to lung cancer.

If we allow the extra language of causation, we need to ask how such an utterance is related to the research at hand. Is there something about research *per se *that demands causal language, along the lines of "X causes Y," be used when describing the results of the research? Or perhaps there is something that is demanded by the need to do the research in the first place, for example determining what caused the disease outbreak at the picnic. Or perhaps there even is something about the culture of epidemiological research (or all scientific research for that matter) that necessitates being able to say X causes Y.

It may appear that a researcher who has a great deal of expertise in an area of research, such as lung cancer and smoking, should be able to say simply and plainly that "smoking causes lung cancer," based on specific research findings. The problem is that the reasons for this "should be able to say" will not be directly supported by the research itself; *it *is not what makes this choice of words compelling. There is no more direct justification for this than for needing to say "I know I have two hands." The person uttering this may also think that they "should be able to say" that this is the case and provide a series of reasons, such as professional expertise, the demands of the piano culture, his comedy routine, etc.

The researcher's need to say "X causes Y" reveals something about his state of mind and his beliefs about what the research shows, but this information is not pertinent to the presentation of the research itself. Thus, we would argue that there are only two states the researcher is left with. If a knowledge or causal statement is uttered without context, whether the playing of the piano, or the setting out of a research process, such a claim is unsupportable. Within the particular context of epidemiological research, the claim is redundant and misleading.

The reader, at this point, may think we are being too harsh on the researcher who has many years of research experience and understands the literature, important alternative hypotheses, etc. All this may be true and the researcher may be contributing important information. The problem comes when the researcher is appealing to only the research itself to support the claim of causation, and the research itself is mute about causation *per se*, although eloquent, hopefully, about how, let's say, smoking increases the risk for lung cancer under specific conditions, controlling for confounders and avoiding biases. There is nothing separate from the results of the research that announces itself as causal. The researcher calling the relationship between smoking and lung cancer a "causal connection" will not be able to point to any element or grouping of elements in the research that unambiguously shows a causal relationship. The researcher can, and even hopefully will, use tools such as analyses of necessary and sufficient conditions, or counterfactual formulations in his research. Nevertheless, even competent handling of tools associated with the search for causal relations does not magically bestow a right to base causal claims on the findings.

### Causal anxiety in the 2004 Surgeon General's report

In the new Surgeon General's report [2], the problem is most easily observed in the scale developed to gauge strength of language used in making causal statements about research. The four-level hierarchy for classifying the strength of causal inferences based on available evidence is as follows (page 18):

A. Evidence is sufficient to infer a causal relationship.

B. Evidence is suggestive but not sufficient to infer a causal relationship.

C. Evidence is inadequate to infer the presence or absence of a causal relationship (which encompasses evidence that is sparse, of poor quality, or conflicting).

D. Evidence is suggestive of no causal relationship.

Different causal methods may be used to choose a particular category among A-D, but no operational criteria for choosing among them is in fact being proposed; the authors instead appeal to a shared notion of what is appropriate in the field. Such an approach is of course fine as long as it is seen for what it is, and is not.

Further, the authors explicitly state that counterfactual claims provide the preferred basis for causal claims: "In this report, the definition of cause is based on the notions of a 'counterfactual' state." [2] (p. 19) Although counterfactuals do provide a powerful approach toward understanding how exposures and outcomes may be related, the specific claim that somehow this particular manual for the proper usage of causal terms might serve as the final arbiter of causal claims is troubling for a number of reasons.

First, there are many causal tools that are important in helping to determine relationships between exposures and outcomes, but what would justify the promotion of this particular approach compared to others? This is a highly provisional claim that fails to account for the fact that counterfactual approaches, like all causal approaches, cannot provide a generally applicable definition of cause (per se) [13]. There are always exceptions and ambiguities that finally point to the undeniable conclusion that methods to determine causal relations are not synonymous with observing objective cause.

Secondly, why does a causal claim need to be made explicit? The authors of the report are quite clear on this when they say, "Without the mantle of 'causal,' the identification of a 'risk factor' does not necessarily carry with it the certainty of disease prevention or delayed onset following exposure reduction or removal." They go on to say, somewhat confusedly, that "the characteristics of evidence that merit calling an association causal involve *extra-statistical *judgments. Because the claim is so central to disease prevention ...." (p. 19). There is a hiatus here between the information in the research findings and the need to come to causal conclusions for the purposes of using these research findings for public health intervention. Although we agree that intervention is an essential part of epidemiological and public health research, it is not clear that being able to say that X causes Y makes any sense, in this regard, except in a very highly contextualized/technical setting. The authors use an ends-justifying-the-means-argument that assumes that explicitly being able to say X causes Y is a necessary element of public health research.

### Telling a good story

Our point is that although it is important to be able to use epidemiological research to predict and intervene at the public health level, to tell the best story possible about the research findings at hand, one doesn't have to say that X causes Y to achieve such an outcome. In fact, one cannot definitively claim such a relationship. We think the approach of the Surgeon General's report is commendable in detailing how one can obtain useful information from epidemiological data. Indeed, showing that smoking, controlling for a host of possible confounders in a cohort setting, increases risk for lung cancer is an adequate causal statement. There is nothing speculative in such a claim; we may accurately describe results in terms of estimates of effects, measures of statistical variance and control of confounders, hopefully replicable, in the best tradition of scientific research. All of this is non-controversial with regard to the practice of epidemiology. There is little room for ambiguity, although one may interpret data in many different ways. But neither of this requires, nor does it support the shift to causal language on the part of the authors; the conclusions and decisions that depend on beliefs about causation can be left to the readers.

When considering how we need to think causally in a public health setting, the salient points involve the usefulness of the information for prediction and possibly intervention. Thus, the usefulness and value of the long, patient description ultimately derives from how well people are convinced that this information provides a basis for some kind of intervention or prediction. Although much has been written about causation, we may, as Sosa and Tooley [12] and Cartwright [1] argue, never be able to have anything but a very specific "singularist" sense of causation; i.e., a sense of causal thinking that is not capable of being generalized with rules or methods, but is insurmountably contextual [8]. Far from being an obstacle, such an approach allows for a great deal more clarity regarding the interpretation of epidemiological research.

As mentioned above, this approach appears to be in line with the work of Parascandola and Weed [5] when they point out that probabilistic models of causation are essentially more flexible than deterministic approaches. Whether discussing determinism in causation or more humble, but no less important, issues about causal tools, there is no need to worry about generalizing the discussion. At best, these tools may act as guides that may make specific research more useful for the purposes of intervention or prediction, without providing access to posited objective causal relationships. That certain contexts, such as legal definitions of what constitutes cause, may force a specific notion of cause to come into play; e.g., as demanded by a rule making body like the office of the Surgeon General, or a Judge, provides no added significance to saying that X causes Y. Clearly, certain contexts may demand a very specific use of causal language. Such technical usage of "cause" etc., perhaps in a deterministic way, as might be demanded by a legal process, will occur in a specific setting. For example, a question such as "how much of the paralysis was caused by the faulty tires?" may be unambiguously germane for the purposes of adjudicating a tort case in which a specific notion of cause is introduced and accepted by all parties. Perhaps such uses of "cause" etc. need this level of description.

In another related example, one might ask, what of the situation when undertaking a marginal structural model (MSM) analysis in which the research differentiates between casual effects and mere effects? Is this not a justified use of cause? The answer is a qualified yes, because such a use is highly defined and limited in its meaning. For the sake of MSM analysis, a causal effect is differentiated from a non-causal effect as a function of how well relevant (a judgment call as to what is relevant) confounders and indirect effects are included in the model. The more complete the more "causal" argument is in regard to alternative hypotheses, the better – i.e., more causal – the model. Crude effects, on the other hand, are those that have included minimal, if any, control of relevant confounding and inclusion of indirect effects. There is no hard-and-fast test of when a mere effect becomes a causal effect. This assessment is up to those doing the research and those who assess it. Thus, what a causal effect seems to actually stand for is a more rigorous analysis. This rigorous analysis will hopefully yield more useful information than a less rigorous analysis in regard to intervention and/or prediction. The work here is not in the naming of something as causal, but in the actual rigor of the analysis. The causal language is thus a shortcut that denotes such rigor. Any foundational causal claims are, in fact, the result of circular reasoning. The main point here is that highly contextualized technical/statistical uses of causal language are not the same as making general causal claims about, for example, smoking causing lung cancer. We are always forced back into asking "under what conditions?"

## Conclusion

We have argued that saying smoking causes lung cancer is either an empty or a redundant statement from a scientific perspective; implicitly or explicitly it belongs in the realm of health policy. Epidemiologists need to be constantly aware of the limits of causal language and also of the demands of making explicit causation claims.

When attention is not sufficiently paid to properly contextualizing causal claims and loosely using causal language, there are potentially real world consequences. For example, cigarette company lawyers were often heard to say that the case has not been definitively made that smoking caused lung cancer. They said this knowing full well that in the real world, there is nothing that can be definitively claimed. Nevertheless, this should not, in any way, be an obstacle for epidemiologists in the role, for example, of expert witnesses, who put forward the strongest possible account of a given research program, such as one that links smoking to lung cancer. Indeed, the best we can hope for here is to make the most compelling case, the most persuasive account, and hope that it will be more, rather than less, convincing. This is not a nihilistic throwing of the baby out with the bathwater. Not being able to say something is definitively causal does not mean that extremely useful information is not available; it is simply not available in the way that is traditionally demanded by this specific research community.

And here we must emphasize that there really is something different about implicit and explicit causal arguments. We can easily defend the claim that a ten-fold risk was found for two pack-a-day smokers compared to those who did not smoke. We simply cite the methods and research findings. We cannot defend the additional explicit claim that this is a causal relationship in the same manner. In fact, trying to justify such a claim results in circular explanations, as seen in the 2004 Surgeon General's report.

Unfortunately, even under the best conditions, we have no control over what rational or irrational processes a person will employ to assess usefulness or causation. For the cigarette executives, at one point in time, almost nothing could have been thought convincing. We could, however, imagine that as the power of the research findings mounted, there would be a decrease in the number of executives actually smoking. This, for us, is a promising kind of convincing causal argument, one that is based in actual changes made in the world. Thus, one could describe a narrative where risk is found to increase 10 times for smokers versus non-smokers and 20 years later tobacco executives were smoking in far reduced numbers. Obviously, similar changes did occur in the general population after the first Surgeon General's report appeared in 1964. There is, however, no final arbiter in this regard. Thus, we cannot create a fail-safe scale, or "causal" regime that will, simply by reaching a certain threshold, result in an uncontroversial notion of "causal" or causation, *per se*.

Efforts to establish standards for making causal claims, as in the new Surgeon General's report, should be encouraged as long as the focus is on developing a more coherent and shared sense of what makes specific research efforts, such as examining lung cancer and smoking, more useful for public health and medical purposes. Explicit causal language, if used in a very technical, agreed-upon sense, such as in the MSM modeling example above, could be similarly useful. But this use of technical causal language, a good use, in our estimation, must be recognized as simply a shorthand for better versus worse analyses, as judged by the author, and not a metaphysical statement about causation *per se *(which is beyond what we can learn from epidemiological findings). Given the difficulties described above, the establishment of an unambiguous meaningful and general notion of causal claims, besides being, for all practical purposes, unavailable, is unnecessary for the real world task of prediction and intervention at the public health level. Thus, claims such as "X is likely to be a causal factor for Y" should only be made if sufficient context and definition is provided, and omitted otherwise. For epidemiology, in particular, and science generally, the devil is in the details.
